# Swift evolutionary response of microbes to a rise in anthropogenic mercury in the Northern Hemisphere

**DOI:** 10.1038/s41396-019-0563-0

**Published:** 2019-12-12

**Authors:** Matti O. Ruuskanen, Stéphane Aris-Brosou, Alexandre J. Poulain

**Affiliations:** 1 Department of Biology, University of Ottawa, Ottawa, ON Canada; 2 Department of Mathematics & Statistics, University of Ottawa, Ottawa, ON Canada

**Keywords:** Environmental sciences, Environmental microbiology, Molecular evolution

## Abstract

Anthropogenic mercury remobilization has considerably increased since the Industrial Revolution in the late 1700s. The Minamata Convention on Mercury is a United Nations treaty (2017) aiming at curbing mercury emissions. Unfortunately, evaluating the effectiveness of such a global treaty is hampered by our inability to determine the lag in aquatic ecosystem responses to a change in atmospheric mercury deposition. Whereas past metal concentrations are obtained from core samples, there are currently no means of tracking historical metal bioavailability or toxicity. Here, we recovered DNA from nine dated sediment cores collected in Canada and Finland, and reconstructed the past demographics of microbes carrying genes coding for the mercuric reductase (MerA)—an enzyme involved in Hg detoxification—using Bayesian relaxed molecular clocks. We found that the evolutionary dynamics of *merA* exhibited a dramatic increase in effective population size starting from 1783.8 ± 3.9 CE, which coincides with both the Industrial Revolution, and with independent measurements of atmospheric Hg concentrations. We show that even low levels of anthropogenic mercury affected the evolutionary trajectory of microbes in the Northern Hemisphere, and that microbial DNA encoding for detoxification determinants stored in environmental archives can be used to track historical pollutant toxicity.

## Introduction

Mercury (Hg) is a naturally occurring toxic metal that is globally distributed because of the volatility of its reduced form [1]. Under anoxic conditions, microbes can transform inorganic Hg into methylmercury, which is bioaccumulated in organisms and biomagnified throughout food webs. Hg is naturally remobilized from geological sources but anthropogenic emissions have dramatically increased since the Industrial Revolution, which took place around the end of the 18th century [2]. As a consequence of anthropogenic activity, the concentration of Hg in the atmosphere is estimated to be almost three times higher than in preindustrial times, and about eight times that of 2000 BCE, when Hg started being used by human civilizations [3]. These estimates are based on biogeochemical models, backed by historical sources [4], archeological evidence [5], and measures of total Hg in sediment cores [6, 7]. However, historical response of ecosystem components to Hg has mostly relied on preserved museum specimens [8], because direct tracking of Hg toxicity and bioavailability in environmental archives, such as ice and sediment cores or permafrost, is currently impossible.

Even though human activities have contributed to increase the amount of Hg in the environment, we do not know how historical Hg deposition has affected key microbial players, which often control the amount of Hg available to food webs [9]. This is a major knowledge gap that potentially hinders policy development, because effective risk reduction strategies depend on a comprehensive understanding of the present and past effects of toxic pollutants on ecosystems [10]. One possibility to track bioavailable Hg through time is to monitor how microbial systems responded to historical toxic Hg levels. The best-known microbial Hg detoxification mechanism, the *mer*-operon, codes for proteins that efficiently detect, transport, and reduce the organic and inorganic forms of Hg to its volatile form, Hg^0^, which then diffuses out of the cell [9, 11]. It is thought that the *mer*-operon evolved millions of years ago, in marine hydrothermal environments under strong geogenic Hg pressure, with subsequent constraints by light, salinity, and redox conditions shaping the evolution of the mercuric reductase MerA (coded by *merA*; [12]). *MerA* is (i) specific to Hg detoxification [13], (ii) omnipresent in the environment [14, 15], and (iii) easily exchanged between coexisting microbial populations via horizontal gene transfers that disconnect this marker from its phylogenetic signal [13]. Therefore, we can expect that *merA* variants be maintained in a given environment based on the selective advantage these variants provide to the community, rather than segregating based on their taxonomy (vertical inheritance). As such, *merA* is an ideal candidate to provide molecular insights into historical exposure to Hg. Previous results suggested an association between *merA* phylogeny and Hg deposition in a remote region [16], but this work was limited in its spatial scope and by the lack of variation in historical Hg deposition. To address these shortcomings, we tested if the evolutionary response of *merA* can be used as a proxy for historical changes in the toxicity, and hence bioavailability, of Hg in sedimentary archives collected over a much broader spatial scale.

Here, we show that regardless of site or deposition rate, the local effective population sizes of *merA* increased at an unprecedented rate starting at the beginning of the Industrial Revolution in the Northern Hemisphere. No such response was observed for our control housekeeping gene, *rpoB*, encoding for the β-subunit of the bacterial RNA polymerase. Our results show that even small changes in Hg loadings can have long-lasting effects on the evolutionary dynamics of microbes detoxifying this pollutant, and that the *merA* gene is a sensitive maker, that can be used to track historical Hg bioavailability over broad, continental scales.

## Material and methods

### Sampling and sample processing

**Fig. 1:**
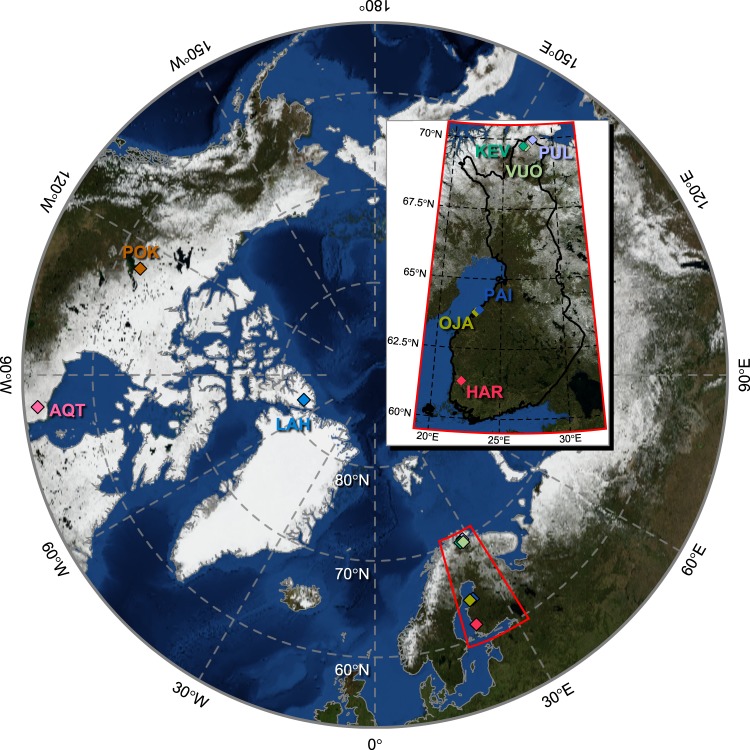
Locations of sampling sites in Finland (blown up in inset) and northern Canada. By alphabetical order: AQT Lake Aquatuk, HAR River Kokemäenjoki, KEV Lake Kevojärvi, LAH Lake Hazen, OJA Lake Öjanjärvi, PAI Lake Päiväjärvi, POK Pocket Lake, PUL Lake Pulmankijärvi, VUO Lake Vuolimuš Cieskuljávri

Intact sediment cores were obtained from eight lakes in Canada and Finland, and a river in Finland, which were selected based on previous data on their Hg deposition history (Table 1, Fig. 1). Low Hg contamination (max [THg] <100 ng g^−1^ dw) sites included Lake Hazen (LAH) in NU sampled in May 2015 in the Canadian High Arctic [17], and Lake Kevojärvi (KEV), Lake Vuolimus Cieskuljavri (VUO), and Lake Pulmankijärvi (PUL) subarctic sites sampled in April 2016 in Finland [18]. To sample moderate Hg contamination (max [THg] from 100 to 1000 ng g^−1^ dw) through long-range transport, we obtained sediment-extracted DNA from a previous study of Aquatuk Lake (AQT), ON, Canada [16], sampled in August 2010; this site also allowed us to validate our original findings using different approaches for quantification and sequencing. To represent freshwater sediments with a history of strong anthropogenic Hg contamination (max [THg] >1000 ng g^−1^ dw), we sampled Pocket Lake (POK), YT, Canada in July 2016, as this lake is under the direct influence of toxic metal deposition from the Giant Mine Au roaster [19]. Lake Öjanjärvi (OJA) and Lake Päiväjärvi (PAI) were sampled in May 2016 in Kokkola, Finland, as they are affected by metal deposition from the Ykspihlaja industrial park [20]. River Kokemäenjoki (KOK) was sampled at the dam reservoir in May 2016 in Harjavalta, Finland. The site has a history of strong Hg pollution from a chlor-alkali plant upstream and a nearby Cu/Ni/Au/Ag smelter [21]. Sizes of their watersheds (if not previously available) were calculated based on the digital elevation models and online tools based on ArcGis (ESRI, Redlands, CA, USA; [22, 23]). Sites in Finland were sampled with an HTH sediment corer [24] (Pylonex AB, Umeå, Sweden) except for Kevojärvi, where a wedge type ice finger sampler (1.5 m) filled with a dry ice/coolant mixture was used to preserve the annual laminae in the sediment. At LAH and POK, a UWITEC gravity corer (Mondsee, Austria) was used. Most of the cores were sectioned at 1 cm intervals in the field within hours of sampling, and were subsampled from the middle of the sections (avoiding the edges) with sterilized tools to minimize cross-contamination and edge deformations in the cores. The sections from Finnish cores were frozen on dry ice or in a −80 °C freezer immediately after sectioning. The POK core was sectioned after sampling with sterilized tools at 0.5 cm intervals, which were frozen at −20 °C. The whole core from LAH was frozen at −18 °C in the field and sectioned later at 1 cm intervals while frozen. Both LAH and KEV sections were then subsampled with sterilized tools from the middle of the sections. No chemical preservatives were used for any samples, all sample containers were sterile, and bleach-sterilized powder-free nitrile gloves were worn throughout sample handling. After the initial freezing, the samples were shipped frozen, stored at less than −18 °C, and thawed only directly before DNA extraction.

**Table 1:** Geomorphological characterization of the sampling sites

Peak [THg]	Site	Abbr.	Sampling coordinates	Lake area	Watershed area	Catchment area to lake area ratio^a^	Mean–max. water depth	References
2739 ng g^−1^ dw	Kokemäenjoki/Harjavalta	HAR	61.33207 °N 22.12895 °E	NA (river)	26100 km^2^	n/a (river)	n/a (river)	[78]
1906 ng g^−1^ dw	Pocket lake	POK	62.50897 °N 114.37377 °W	0.048 km^2^	0.095 km^2^	1.98	2 m–6 m	[79, 80]
1057 ng g^−1^ dw	Öjanjärvi	OJA	63.80657 °N 22.99994 °E	12 km^2^	^b^3970 km^2^	331	1.6 m–9 m	[81]
415 ng g^−1^ dw	Päiväjärvi	PAI	63.88923 °N 23.24727 °E	0.13 km^2^	^c^0.95 km^2^	7.31	n/a–1.8 m	[82]
110 ng g^−1^ dw	Aquatuk lake	AQT	54.3281 °N 84.5686 °W	8.28 km^2^	^c^231 km^2^	27.9	n/a–12 m	[83]
126 ng g^−1^ dw	Pulmankijärvi	PUL	69.97459 °N 28.00415 °E	11.2 km^2^	800 km^2^	71.4	11 m–34 m	[84–86]
91 ng g^−1^ dw	Kevojärvi	KEV	69.75804 °N 26.99785 °E	1.1 km^2^	1470 km^2^	1340	11.1 m–35 m	[87]
63 ng g^−1^ dw	Lake Hazen	LAH	81.8245 °N 70.71604 °W	540 km^2^	6860 km^2^	12.7	95 m–267 m	[17, 88]
50 ng g^−1^ dw	Vuolimus Cieskuljavri	VUO	69.73197 N ° 27.09612 °E	0.39 km^2^	^c^22.5 km^2^	57.7	n/a–1.9 m	[89]

^a^Calculated based on lake and watershed area

^b^Watershed area combined with hydrologically connected Luodonjärvi (area 73 km^2^, mean depth 2.6 m, max depth 11 m)

^c^Estimated in this study

### DNA extraction, chemistry, and dating

Samples from the cores were homogenized, and DNA extraction was done in duplicate from 0.25 g (wet weight) subsamples of each section. The subsamples were first washed with a buffer (10 mM EDTA, 50 mM Tris-HCl, 50 mM Na2HPO4·7H2O at pH 8.0) to remove PCR inhibitors [16, 25]. Environmental DNA, consisting of nucleic acids from alive, dormant, and dead organisms in the sediment, was extracted with the MOBIO PowerSoil® DNA Isolation Kit (Carlsbad, CA, USA) and the duplicate extracts were combined. Duplicate negative control extractions were made from the wash buffer to assess the kit contamination and combined after extraction.

For chemistry and dating, the core horizons were freeze dried and their water content was measured. THg data for the AQT samples were obtained from a previous study [16], and in other cores it was quantified based on the thermal decomposition, gold amalgamation, and atomic absorption with a MA3000 mercury analyzer (Nippon Instruments Corporation, College Station, TX, USA) at the Laboratory for the Analysis of Natural and Synthetic Environmental Toxins (LANSET, University of Ottawa, ON, Canada; see SI archive for primary measurements). Marine sediment certified reference materials MESS-3 and MESS-4 (National Research Council of Canada), and Buffalo River Sediment NIST-2704 (National Institute of Standards and Technology) were used as calibration controls for [THg] measurements.

Annually laminated (i.e., varved) sediments deposit in Kevojärvi, which enabled dating these sediments in high resolution by varve counting. The dating for the Kevojärvi core in the current study (KEV) was calibrated to a parallel core (KEVO-1) dated earlier by a highly resolved ^137^Cs profile, where the peaks from the radioactive fallout from Chernobyl accident in 1986 and the 1960’s nuclear testing were used to adjust the varve chronology (E. Haltia, University of Turku, personal communication, February 2019). The ^210^Pb and ^137^Cs dating of the other cores for the current study (VUO, PUL, PAI, OJA, KOK, and HAR) was completed with an Ortec High Purity Germanium Gamma Spectrometer (Oak Ridge, TN, USA) at LANSET. Certified reference materials obtained from International Atomic Energy Association (Vienna, Austria) were used for efficiency corrections, and results were analyzed using ScienTissiME (Barry’s Bay, ON, Canada). Constant Rate of Supply models were used for all primarily dated cores, and cross-calibrated against the peak in ^137^Cs (see SI archive). The peak was estimated to occur at 1986 in the Finnish cores (VUO, PUL, PAI, OJA, KOK, and HAR) as deposition from the Chernobyl disaster [26]. Geochronology data from previous studies was used directly for the AQT core [16], and the LAH and POK cores, where the profiles were adjusted for different sampling times. The core from LAH was obtained from the same exact site with the same equipment as a previously ^210^Pb-dated core [27] and its sedimentation-adjusted dating was used for the new core. For POK, the ^210^Pb dating of a previously analyzed core [19] was used, since we observed no differences in the THg profiles of the cores (*P* = 0.58; Fig. S1). For individual horizons in the cores with no direct ^210^Pb measurements, dates were estimated either by interpolating between measured horizons or extrapolating the models by fitting a second order polynomial to the modeled dates (all *R*^2^ > 0.97; Fig. S2). Extrapolations were only performed down to 1750 CE, since sediment chronology can be underestimated below supported ^210^Pb values [28]. We addressed the potential issue of the mobility of DNA and Hg within the sediment profiles in a previous study [16].

### Gene quantification with droplet digital PCR (ddPCR)

Copy numbers of *merA* and *glnA* were quantified from all DNA samples (*n* = 126; Fig. S3) with the Bio-Rad QX200 ddPCR system, and the primary data were processed with the QuantaSoft suite (Bio-Rad, Hercules, CA, USA). Briefly, droplets were generated from PCR reactions according to the manufacturer’s guidelines and the reactions were run according to conditions outlined in Table S1, using primers for *merA* developed in-house and for *glnA* as in [29]. ddPCR enables the quantification of target sequences in samples, similarly to quantitative/real-time PCR, but with simplified workflow, and increased accuracy and precision compared with other quantification methods [30]. The accuracy of the assay was controlled with previously quantified plasmid templates containing the target gene for both *merA* and *glnA*, and elution kit buffer extractions as negative controls. The baselines for positive droplets in each sample were adjusted based on the negative and positive controls. The copy numbers (and 95% CI) for both genes were then normalized to copies per ng DNA in each sample.

### Amplicon sequencing

The samples were screened for the presence of *merA* and *rpoB* with conventional PCR (Table S1). The primers for *merA* had been designed to target Proteobacteria and Firmicutes [31] and to specifically contain the motif for the terminal cysteine residues in the *merA* gene. The primers for *rpoB* were designed for this study with a broad range of specificity (notable exclusions: Cyanobacteria, Gracilibacteria, Microgenomates, and SR1). Despite our best efforts (e.g., fresh reagents and enzymes, changing laboratory space, and equipment), negative controls sometimes were positive for *merA* (Fig. S4), but never for *rpoB*, which might have been due to the presence of contaminating DNA in the reagents [32, 33]. We focused on the single-copy housekeeping gene *rpoB* as a control instead of *glnA* because it enabled the design of a PCR amplicon similar in size to *merA* (and longer than with *glnA*, which proved too short for diversity analyses). Furthermore, *rpoB* is also a marker gene for bacterial phylogeny [34], which is useful for discerning between the variation in overall microbial community structure and variation specific to the *merA* Hg resistance gene. Thus, we sequenced 30 samples and a kit negative control for *merA*, and 30 samples for *rpoB* (Fig. S3) with Illumina MiSeq (paired-end 250 bp) at Génome Québec (Montreal, QC, Canada).

### Sequencing data processing

All processing was completed similarly for both *merA* and *rpoB* unless otherwise stated. The reads were paired with pear v0.9.10 [35] with >100 bp overlap and the stringency set to *P* = 0.0001. The primers and barcodes were removed, sequences were truncated to the first ambiguous base call, or where Phred scores fell <28 in a 2 bp-window. Sequences shorter than 150 bp were removed with QIIME 1.9.1 [36]. Chimeric sequences were removed with vsearch v2.0.0 [37] utilizing the uchime algorithm [38] against databases of 614 *merA* or 286 *rpoB* sequences truncated to the amplified region (gene databases are included in the SI archive). To filter nontarget reads, the sequences were translated to amino acids with EMBOSS 6.5.7 [39], searched against custom HMMs (included in the SI archive) constructed from the *merA* and *rpoB* databases with HMMER 3.1b2 [40], and sequences not matching the profiles (*merA*: *E* > 10^−25^; *rpoB*: *E* > 10^−5^) were removed. The nucleotide sequences were then dereplicated and clustered with Swarm 2.1.9 [41], and variant abundances were summarized with custom Perl and R scripts (see SI archive). The sequences were subset to cluster seeds, aligned with Muscle [42] through TranslatorX [43] to find a common reading frame, and trimmed to the first aligned codon position. The *merA* sequences that did not encode a Tyr605/Phe605 residue required for activity [13] were removed, and sequences that bridged gaps in the alignments (except for positions after 343 bp for *rpoB*) with <10% occupancy were removed in both *merA* and *rpoB* alignments. The sequences were realigned with MAFFT [44] through TranslatorX, trimmed with trimAl 1.2rev59 [45] using the ‘-gappyout’ option, and sequences still containing gaps were removed.

Out of 2580 quality-controlled *merA* variants in the samples, 37 (1.4%) were also present in the sequenced negative control (kit extract). These were first removed (Fig. S5), and the remaining read coverage was assessed over estimated calendar dates (Fig. S6). Maximum likelihood trees were constructed from the sequences that passed all quality control steps (*n* = 2,551) with FastTree 2.1.9 [46] using the GTR + Γ model of sequence evolution [47]. Long branches, potentially indicating rogue sequences, were then removed with TreeShrink 1.0.0 [48] using a false positive error rate of FDR = 0.01, and the trees were re-reconstructed with FastTree under the same model as before.

### ddPCR data analyses

For all ddPCR analyses, the data were limited to samples with estimated dating >1750 CE, and the HAR samples were removed because of the mixed core profiles. Briefly, the correlation of gene copy numbers of *merA* and *glnA* with [THg], dating, and sediment depth (distance from surface of the sediment) were assessed with linear mixed-effects models using ‘lmer’ from lme4 [49]. The copy numbers were ${\log}_{10^{-}}$transformed (after assessing the normality of residuals), all variables were scaled and mean-centered, and sampling site was used as a random effect (random intercept). Also, identified outliers (see Fig. S3; Pulmankijärvi, points around 1900 CE for *glnA*) were removed from the final model. The significance of model variables was assessed from the transformed 95% confidence intervals, and the effect sizes were estimated over their range from model predictions. To compare our ddPCR data against previous studies of *merA* copy numbers across [THg] [50, 51], we averaged and ${\log}_{10^{-}}$transformed the normalized copy numbers of *merA*, and averaged [THg] over the top 5 cm of sediment samples at each site, and investigated their correlation across the sites with a least square linear regression.

### Diversity analyses

Abundances of *merA* and *rpoB* variants within each samples were normalized with cumulative sum scaling with metagenomeSeq [52]. Patterns in beta-diversity (between-sample diversity) of the genes were analyzed with a Double Principle Coordinate Analysis using phylogenetic distances of the variants followed by non-metric multidimensional scaling (NMDS) on the sample distance matrix with phyloseq [53]. Influence of [THg] (${\log}_{10^{-}}$transformed ng g^−1^ dw), date (CE), and sampling site on the patterns observed in the ordinations were assessed with the functions “ordisurf” and “factorfit” in vegan [54].

### Bayesian demographic reconstructions

To reconstruct the phylogeny and past population dynamics at the studied lakes, the *merA* and *rpoB* variants were first subset per sampling site and variants present in more than one sample per site were removed to eliminate cross-contamination. If there were more than 50 variants in a sample, they were reduced to the most phylogenetically diverse variants by calculating the patristic distances with ape [55], followed by Ward’s hierarchical clustering [56], and cutting the tree to 50 groups. The timed phylogenies of *merA* and *rpoB* were reconstructed for each lake with BEAST v1.8.0 [57] using the GTR + Γ model of sequence evolution [47], with a skyline prior on the speciation process, and an uncorrelated lognormal prior on rates [57] (mean = 0.001, stdev = 1.0); an additional lognormal prior was placed on tree heights (mean = 10, stdev = 1.0, offset = 500 years); chains were started from UPGMA trees. Markov chain Monte Carlo samplers were run in duplicate for up to two billion generations (or until convergence), using a thinning of 20,000 to decorrelate samples; converged duplicate runs were combined after removing burn-in periods (between 5 and 35% of each chain length, according to the mixing of each chain). The maximum *a posteriori* trees were summarized from the combined collections of trees for each site and gene with TreeAnnotator v1.8.0 [57]. Due to computational limitations, the POK combined trees were thinned to half the sampling frequency of other sites for calculating the summary tree. The association between deposition date of the sample and phylogenies of the gene variants in each lake was tested with BaTS [58] using 10^3^ replicates on a subsample of 10^4^ trees sampled from the posterior distributions;[16] *P* values were based on the Association Index [59]. The ancestral demographics estimated with effective population size (*N*_*e*_) were reconstructed through Bayesian Coalescent Skyline plots using 25 intervals and piecewise-constant spline regressions [60].

### Random forest modeling of *N*_*e*_ and breakpoint analysis

To model the overall trends in the demography sizes of both genes, random forests were grown to 5000 trees with ranger [61]. The median of the estimated *N*_*e*_ was used as the dependent variable with ^210^Pb/^137^Cs dates (CE) and sampling sites as predictors (Fig. S7). To assess the stability of model predictions, the data were randomly split ten times while taking care of class imbalance among sites. In the splits, 80% of the data were used for the training of each model and 20% for testing. Partial dependence of model predictions on the predictors was analyzed in each of the ten models with edarf [62]. The effect of date was important for the accuracy of model predictions for both genes: a sensitivity analysis showed a decrease in the pseudo-*R*^*2*^, when date was omitted from the models, on average from 0.96 to 0.41 for *merA* and from 0.95 to 0.80 for *rpoB*. The alternative models where [THg] was added as a predictor had similar performance and results to the main models (Fig. S8), but with a slightly delayed onset of the increase in *merA N*_*e*_ and earlier onset for the increase in *rpoB N*_*e*_. However, the prediction accuracies of the alternative models, or their results, did not change with the addition of [THg]. Also, its values were imputed (by the overall mean) for missing dates, i.e., before the lowest extent of the [THg] measurements in each core. Thus, we chose the parsimonious model without [THg]. Finally, segmented regression analyses were performed on both the *merA* and *rpoB* partial dependence plots of the relationship between date and estimated *N*_*e*_ with segmented [63], based on a single breakpoint and a starting value of 1800.

## Results and discussion

### Total mercury concentrations affect *merA* diversity but not its abundance

To understand how microbes responded to different levels of anthropogenic Hg deposition, we collected sediment cores from eight lakes in Canada and Finland, as well as a river in Finland (Fig. 1).

We first tested if the abundance of *merA* (quantified with ddPCR) correlated with total mercury concentration ([THg]), sediment deposition date, or sampling depth (from sediment surface) in these sediment cores (Fig. S3). We assessed these results against a single-copy housekeeping gene, *glnA*, as our first control gene. We found that the copy numbers of *merA* did not change with sediment depth, nor the estimated deposition date, or even with [THg] (Fig. S9a). However, the number of *glnA* copies, our control gene, decreased with sediment depth (Fig. S9b, c). These results contrast with previous studies reporting an increase in *merA* copy numbers over long-term Hg contamination of various soils [50] or with bioavailable Hg in snowpacks [51] (Fig. S10). However, such studies focused on spatial variation, and did not address the changes over time—or geochemical gradients—at each location.

**Fig. 2:**
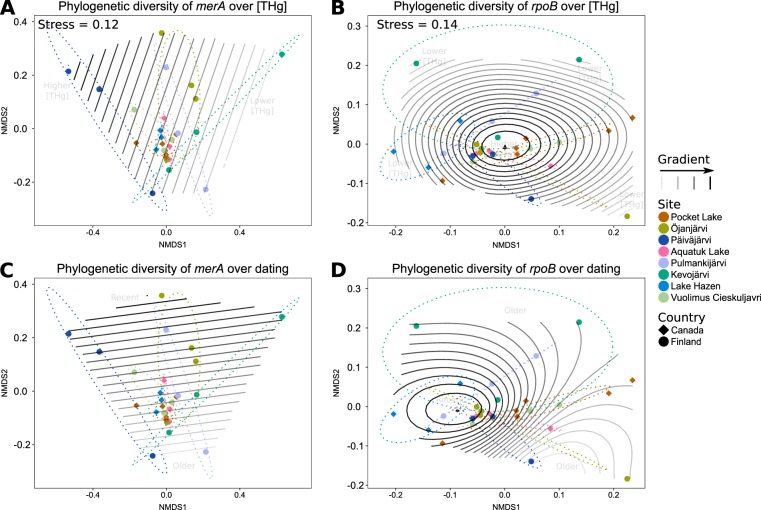
Beta-diversity of *merA* and *rpoB*. Correlations of the NMDS ordination with [THg] (**a**, **b**) and dating (**c**, **d**). Samples from the same site are color-coded and grouped with ellipses. Stress values for the NMDS ordinations are indicated in **a** and **b**, and are identical for **c** and **d**, respectively. All effects tested by the general additive models for [THg] and dating (shown as gradients) were significant (*P* < 0.01), and the values of each variable increase toward the solid lines. The directions of the gradients are further annotated in the plot area with gray text (higher/lower [THg], and recent/older dates)

Given the lack of variation in the abundance of *merA* along [THg] gradients, there could instead be changes in its genetic diversity, suggesting the adaptation to increasing Hg selective pressure. To address this point, we compared the diversity of *merA* sequences among samples (beta-diversity), both over varying [THg] and temporal gradients in the sediment cores. In this case, we used an additional control gene, the single-copy housekeeping gene *rpoB*, encoding for the RNA polymerase β-subunit, to provide more accurate evolutionary information; indeed, *glnA* amplicons proved too short (<156 bp) for this subsequent step of the analysis. These analyses showed that *rpoB* variants were similar among sites (*P* = 0.09) and countries (*P* = 0.88). On the other hand, *merA* variants differed significantly among sites (*P* = 0.01), while countries showed similar variational responses (*P* = 0.30; Fig. 2). This pattern suggests that *merA* diversity is affected by [THg] gradients, contrary to *rpoB*. To confirm this observation, we fitted General Additive Models (GAM) of [THg] and dating on the ordinations of *merA* and *rpoB* diversity. All these fits were significant (*P* < 10^−17^), with *merA*’s beta-diversity correlating linearly with both [THg] and temporal (dating) gradients. The diversity of *rpoB* failed to show any significant linear response to any of these gradients, only exhibiting phylogenetic similarity at the sediment surfaces (Fig. 2).

These contrasted results suggest that the two genes, *merA* and *rpoB*, have responded differently to an increased Hg deposition over time. As *merA* codes for a protein (MerA) whose sole known function is to detoxify Hg, it can be posited that *merA*’s evolutionary trajectory has been affected by historical changes in Hg deposition.

### Swift evolutionary response of *merA* from the onset of the Industrial Revolution

**Fig. 3:**
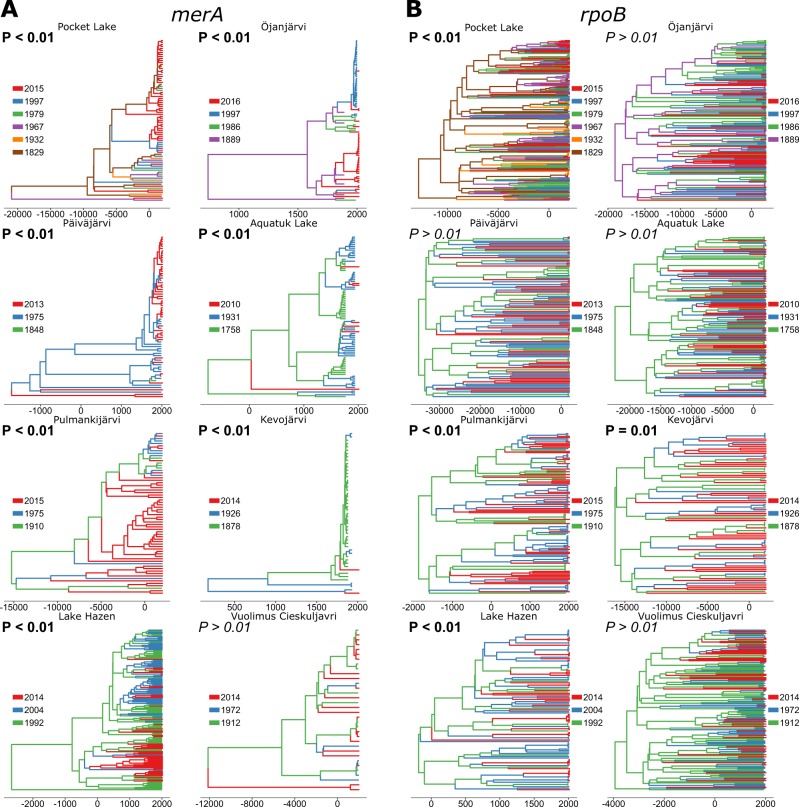
Maximum *a posteriori* trees from the relaxed molecular clock analyses. Trees are shown for *merA* (**a**) and *rpoB* (**b**) at each site. Sampling horizons were color-coded as shown next to each tree and are identical across trees for the topmost three horizons. The *x*-axes are in unit of CE years

To test this hypothesis, we reconstructed both the phylogeny and past demographics of microbes carrying *merA* genes with Bayesian relaxed molecular clocks [64]. First, the trees constructed for *merA* showed a significant association (*P* < 0.01) with the sediment horizon (i.e., depth/date, as a categorical variable) in all lakes except for Vuolimus Cieskuljavri, which may be due to its low [THg] (max [THg] = 50.4 ng g^−1^ dw; Fig. 3).

This clustering of phylogenetic clades by dates not only argues against stratigraphic leakage of both *merA* and Hg, but also suggests a rapid evolutionary turnover, where *merA* gene variants are replaced from one horizon to the next. Critically, such a high turnover was however not observed for our control gene, *rpoB*, where this association between sediment horizon and phylogeny was significant in only three out of eight (38%) sites.

Even if the significance of this gene-specific association of clades with dates cannot be rejected (Binomial test, *P* = 0.73), *rpoB* is considered to be a reliable phylogenetic marker gene in bacteria [34] and such associations might reflect the structuring of the microbial communities in sediments in response to in-situ physical and geochemical gradients developing over time in surface sediments (e.g.,[65]). Because beta-diversity patterns inferred for *rpoB* differed from that of *merA*’s, the phylogenetic signal recorded in each gene seems to be driven by different processes at the microbial community level. This lack of a clear signal prompted us to go beyond a mere phylogenetic assessment, and to further evaluate how Hg delivery affected the evolutionary dynamics of the two genes.

**Fig. 4:**
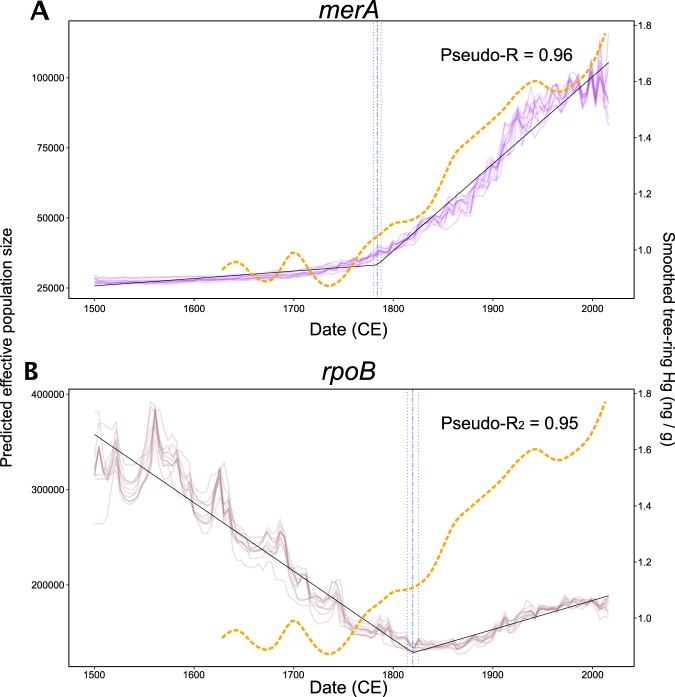
Partial dependence plots of predicted effective population size as a function of calendar date for *merA* (**a**) and *rpoB* (**b**). Results are shown from fitting ten randomized models for each gene. The pseudo-*R*^*2*^ scores quantify average prediction accuracy of the models, which also included the sampling site as a variable. The black lines show the segmented linear model fits and the blue lines indicate their breakpoints (dot-dash lines) surrounded by their 99% CI (dotted lines). Dashed orange lines show the smoothed atmospheric Hg concentrations in subarctic Canada and were redrawn using data from [73] (color figure online)

Our reconstructions of the *merA* demographics based on Bayesian skylines showed sigmoidal increases in the scaled effective population sizes (*N*_*e*_) of this gene over time at most sites, matching the trends in [THg] (Fig. S11a). In contrast to *merA*, the *N*_*e*_ of *rpoB* varied widely (Fig. S11b) and did not seem to coincide with any trends in [THg] at any of the sites, which is consistent with the results of our GAM analyses (Fig. 2). Finally, to average over these site-specific responses, we fitted random forest models to the reconstructed demographics for each gene, over all the sampled sites (Fig. 4). We found that *merA*’s demographics initially exhibited a slow increase of *N*_*e*_, followed by a sharp increase through time (pseudo-*R*^2^ = 0.96; Fig. 4a). Again, these trends were not observed for *rpoB* (pseudo-*R*^2^ = 0.95; Fig. 4b). To determine the dates at which these demographic dynamics changes occurred, we fitted segmented regressions of the partial dependence of *N*_*e*_ *vs*. date.

For *merA*, we estimated that the reconstructed paleodemographics changed around 1783.8 ± 3.9 CE (99% CI; adjusted *R*^2^ = 0.98, Fig. 4a), date after which the rate of increase rose by an order of magnitude. The latest global increases in Hg deposition originated from the increased wood burning in North America [66] at the end of the 18th century, and coal combustion fueling the Industrial Revolution in Europe, which began around 1760 [67, 68]. For comparison, we overlaid atmospheric Hg^0^ concentrations from high-resolution tree-ring data in subarctic Canada [69] on our reconstructed demographics of *merA* (Fig. 4a). The temporal coherence of *merA N*_*e*_, derived solely using sediment DNA, and that of atmospheric Hg^0^ levels, is striking, and independently supports an increase in Hg selective pressure. Our results show that across our sites, located up to 5500 km apart in the Northern Hemisphere, historical anthropogenic Hg emissions affected the evolutionary dynamics of *merA*—allowing us to track variations in Hg toxicity, and hence bioavailability, through time. The magnitude of the evolutionary response of *merA* to an increased Hg deposition was likely related to changes in bioavailable Hg causing a selective pressure on *merA*, rather than solely to changes in [THg]. Indeed, the total concentration of a metal is rarely a good predictor of its toxicity, and this is true for Hg [13]. Rather, metal speciation, i.e., the complexes that a metal forms with inorganic and organic ligands in a solution and at interfaces, affects how metals interact with living cells, causing toxicity [70]. Only total Hg concentrations, and not Hg bioavailability or toxicity, can be directly determined from environmental archives. As environmental conditions change over time, we can expect that microbes present in the water column or in surface sediments be exposed to different levels of Hg exhibiting varying bioavailability and toxicity, and record these variations in selective pressures in their genomes. Furthermore, Hg bioavailability might differ among sites due to differences in local geochemistry affecting Hg speciation (e.g., [dissolved organic matter]; [71, 72]). Such local difference can be observed for POK and Öjanjärvi (Fig. S11a).

For the control gene *rpoB*, the reconstructed paleodemographics were highly variable compared with *merA*, decreasing since the 1500s, and started to increase around 1819.6 ± 5.6 CE (99% CI; adjusted *R*^2^ = 0.93; Fig. 4b). Unlike *merA*, for which Hg concentration and speciation are drivers that can directly explain its demographics, the driving forces behind *rpoB N*_*e*_ remain unclear, and are likely plural and complex. These *rpoB* demographics reconstructions need to be interpreted carefully. Indeed, as previously mentioned, and contrary to *merA*, our GAM analyses determined that there was no significant linear association between *rpoB* diversity and depth or time, and the Bayesian relaxed molecular clocks only supported the clustering of *rpoB* phylogenetic clades by dates in three of the studied lakes. Contrary to *merA*, it is more likely that the changes in *N*_*e*_ of *rpoB* were caused by a restructuring of the sediment microbial communities driven by in-situ geochemical gradients, as implied by our beta-diversity results. Whether or not *N*_*e*_ of *rpoB* also responded to global drivers of change (e.g., temperature, pCO_2_), in addition to local drivers, remains to be tested. Here, the critical point is that, irrespective of what the drivers of the evolutionary dynamics of *rpoB* are, changes in *rpoB*’s *N*_*e*_ are not driven by historical changes in Hg loadings, and strongly contrast with the paleodynamics reconstructed for *merA*.

The evolutionary effects of an increase in Hg loadings on *merA* were sensitive, rapid, and long lasting. These observations warrant continued monitoring of the sites sampled in this study as stricter emission regulations are being implemented. Additional sampling sites should cover the Southern Hemisphere and sites that have experienced sustained and extreme historical Hg levels. However, sites experiencing high Hg pollution, such as Kokemäenjoki/Harjavalta, need to be chosen carefully, as they are often heavily mined or subjected to remediation strategies involving physical alteration of soil or sediments; such physical alterations render them unsuitable for dating and subsequent demographic reconstructions. Finally, to support our field observations, laboratory experimental evolution approaches will be useful to hone the evolutionary models and test for the reproducibility of their trajectories [73].

With implementation of the Minamata convention underway [74], gaining insights into historical responses of biota to anthropogenic Hg is critical. Indeed, a recent study showed divergence between Hg levels in aquatic wildlife and atmospheric values, especially in the last two decades [75], that might be caused by climate change. The overriding and opposite effect that climate change may have on a reduction of Hg emissions requires that governments and environmental agencies worldwide be equipped with the means to accurately assess the efficiency of the reduction of Hg emissions on ecosystem health. In addition to the ongoing efforts to track the loss of eukaryotic biodiversity and habitat [76], equal if not greater scrutiny should be paid to the concurrent changes in the distribution, abundance and activities of microorganisms affected by the consequences of anthropogenic activities. In this study, we highlighted how the evolutionary dynamics of a discrete microbial gene have responded to human activities. We posit that our findings can be applied to any globally distributed contaminants for which microbes have evolved specific detoxification determinants. In the near future, it is reasonable to think that improved sequencing throughput and advances in computational techniques, which now allow for the recovery of thousands of genomes from environmental metagenomes [77], will help determine the paleodemographics of multiple genetic determinants simultaneously. This would provide much needed genome-level information on the historical response of microbes to ongoing anthropogenic environmental changes.

## Supplementary information

Supplementary Information

## Data Availability

All code and primary data in this study are available through GitHub (https://github.com/Begia/merA-evolution/). The raw sequence data and the quality-controlled *merA* and *rpoB* variant sequences have been submitted in NCBI repositories. These data are stored and can be accessed under the bioproject ‘PRJNA539962’ (http://www.ncbi.nlm.nih.gov/bioproject/PRJNA539962).
