# Supplementary material for: Swift evolutionary response of microbes to a rise in anthropogenic mercury in the Northern Hemisphere
Source: ISME J. 2019 Dec 12;14(3):788–800. doi: 10.1038/s41396-019-0563-0 (PMC7031220; doi:10.1038/s41396-019-0563-0)
Supplement: Supplementary file 1 — Supplementary Information [file 41396_2019_563_MOESM1_ESM.pdf]

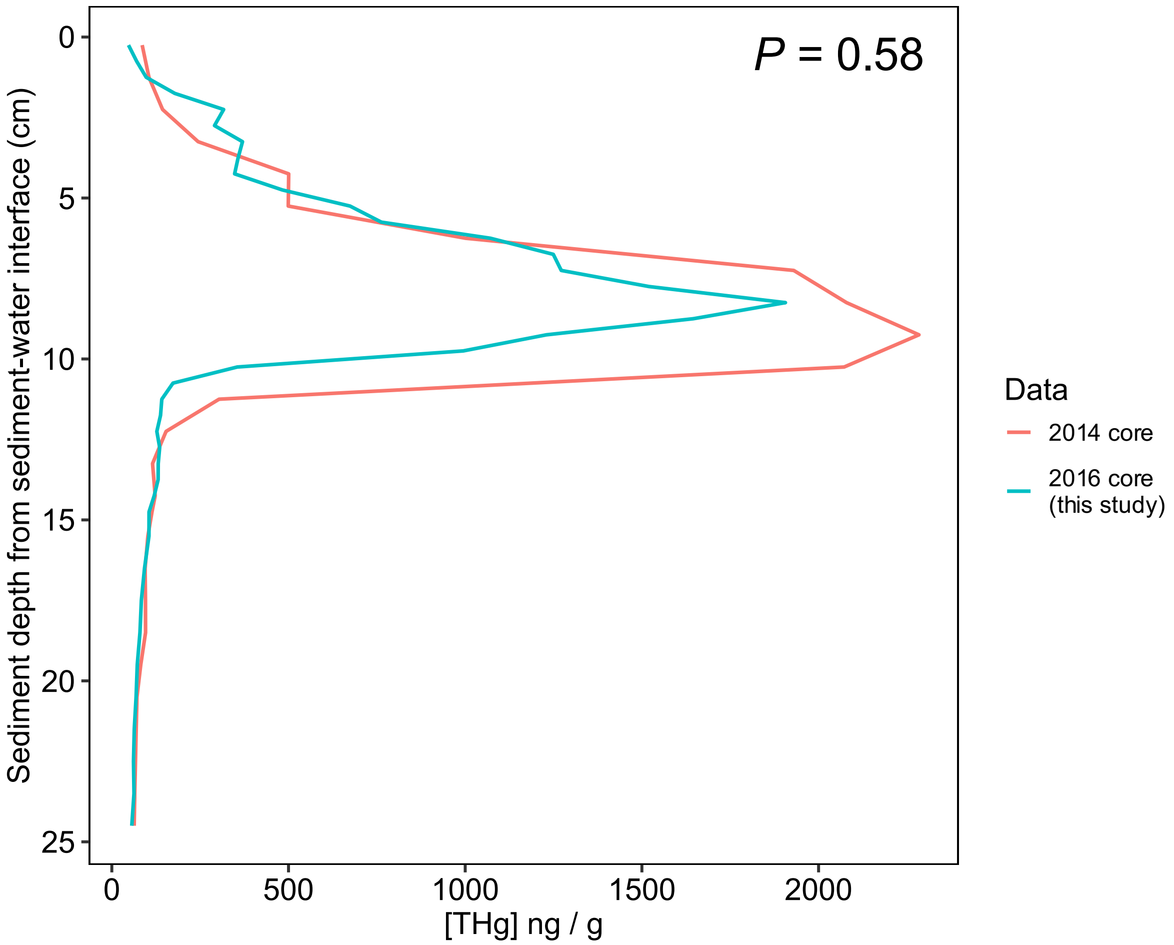

Figure S1. DNA extraction, chemistry and dating. Comparison of previously published data of [THg] in a sediment core from Pocket Lake (red line; Thienpont et al., 2016) to [THg] data from the core analyzed in the current study. The *P*-value is based on the Kolmogorov-Smirnov test.

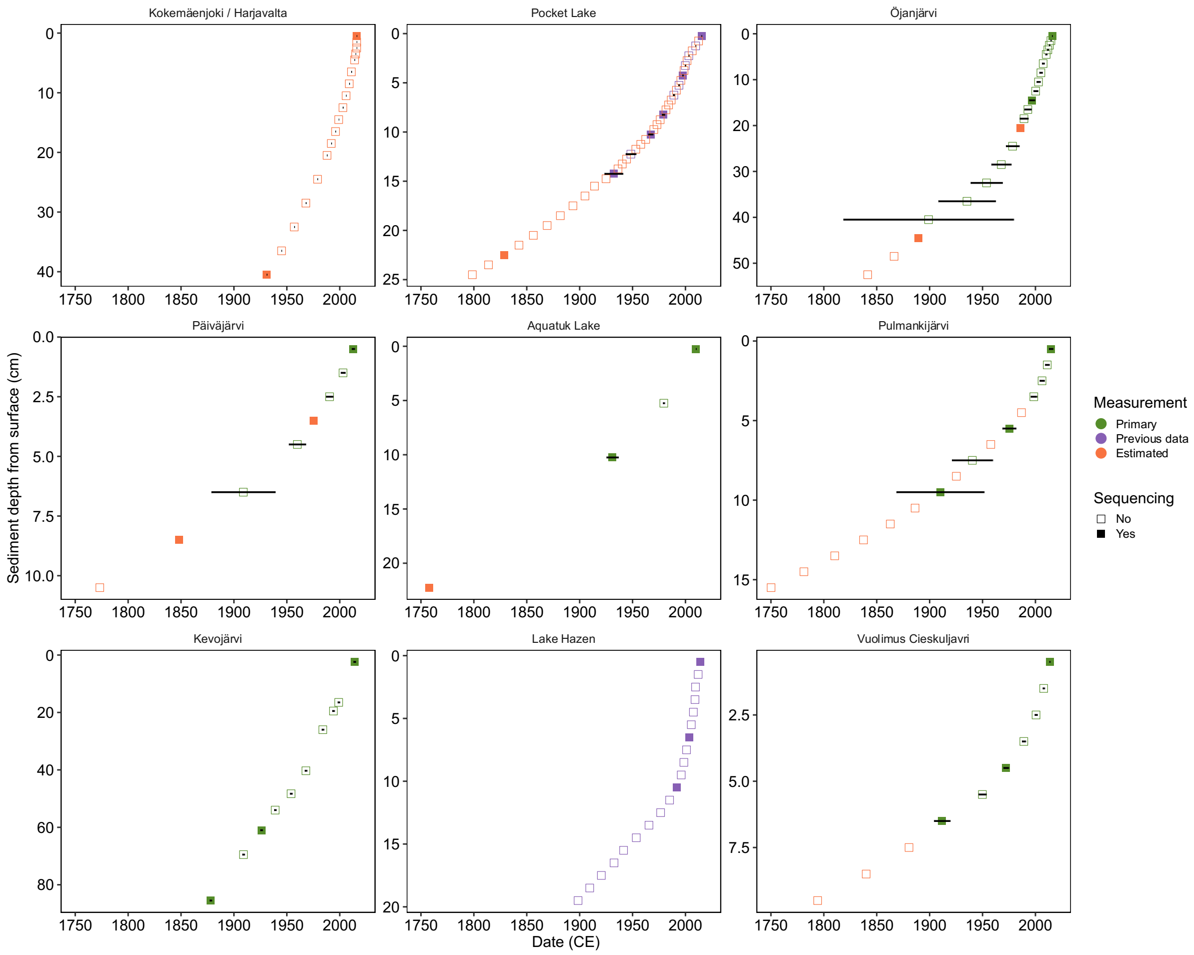

Figure S2. Dating profiles of sediment cores examined in the current study. Samples used for high-throughput sequencing of the *merA* and *rpoB* genes are shown as filled squares. All fits of second order polynomials to the measured dates, used in the extrapolations, had $R^2 > 0.97$. The ^{210}Pb profile of the Kokemäenjoki / Harjavalta core appeared to be mixed; the dating shown here, without error bars, was not used in subsequent analyses.

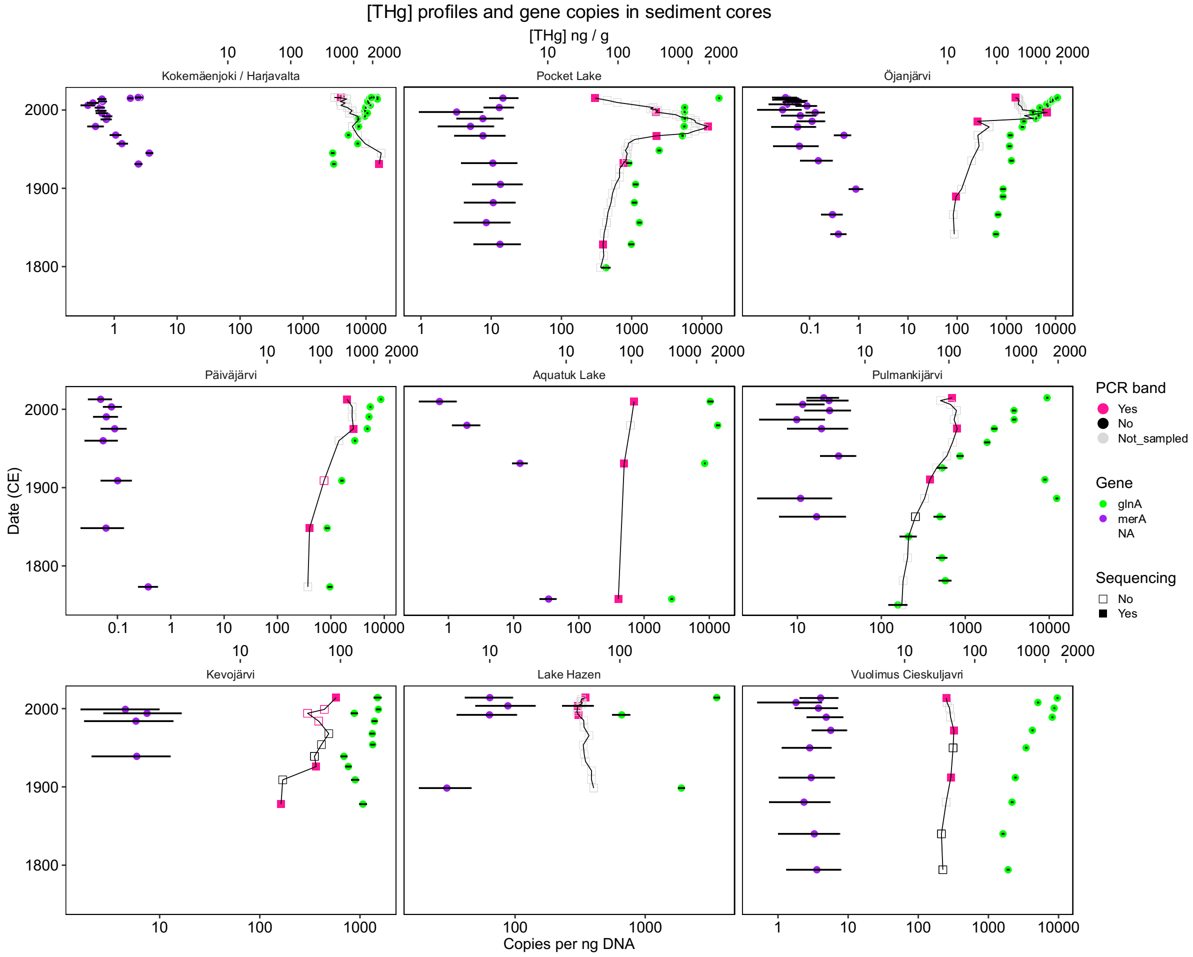

Figure S3. Dated gene quantification with droplet digital PCR and amplicon sequencing. Total Hg profiles and gene copy numbers in of *merA* and *glnA* the sediment cores. The samples that were sequenced are shown as solid squares. CE dates were ^{210}Pb-derived.

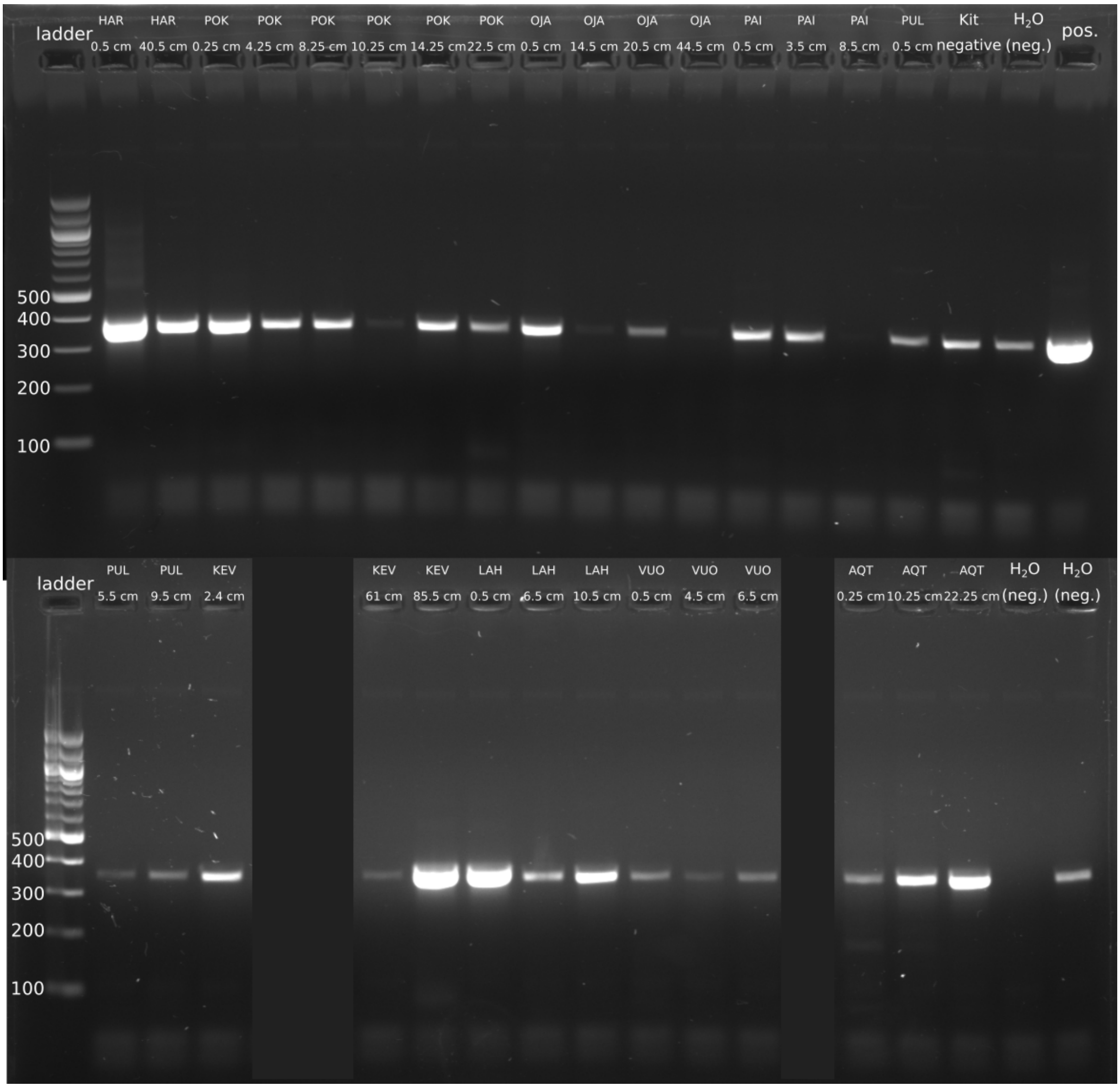

Figure S4. Amplicon sequencing. PCR screening of the samples selected for sequencing of the *merA* gene using first the NlfF/NlfR primers followed by the NsfF-CS1/NlfR-CS2 primers. The reaction conditions are outlined in Table S1. Electrophoresis was performed in a 1.5% agarose gel in a 1× Sodium-Borate buffer with 0.5 µg mL-1 EtBr at 11V / cm for 30 min. NEB QuickLoad Purple 100 bp ladder was used (5 µL) and 10 µL of each sample + 2 µL loading dye was loaded. The positive control was a Tn501 plasmid containing the *mer*-operon.

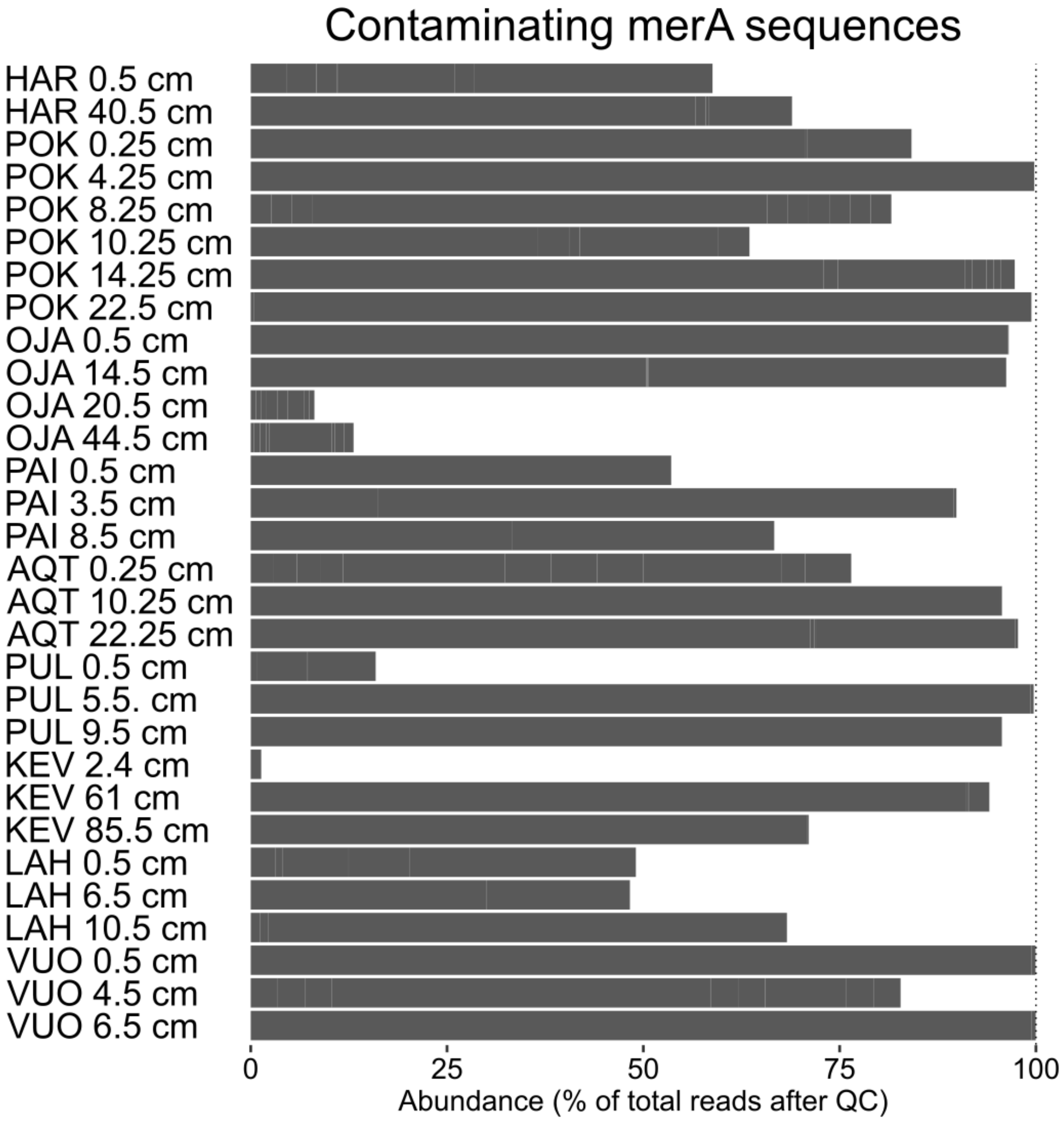

Figure S5. Sequencing data processing. Proportion of total reads in the samples identified as contaminants (any *merA* variants present in the negative control).

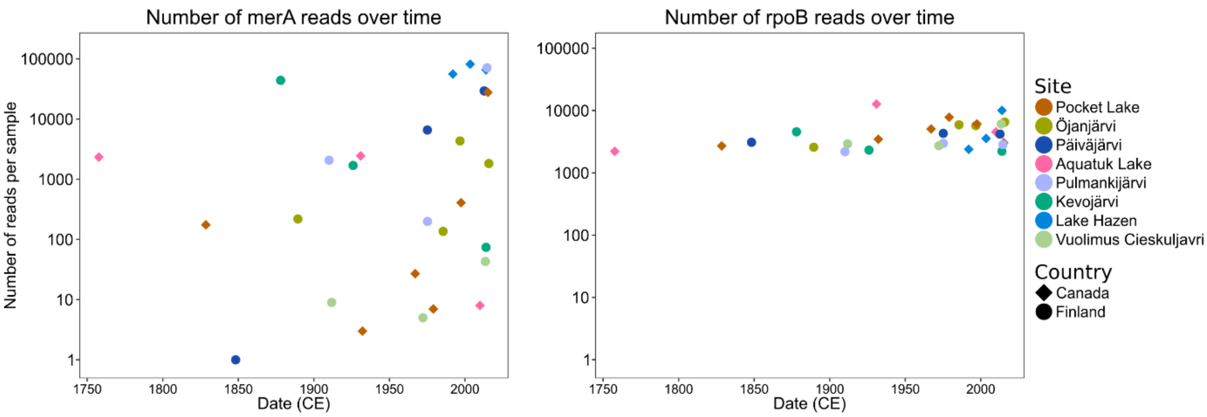

Figure S6. Sequencing data processing. Number of *merA* and *rpoB* reads per sample after quality control, with estimated calendar dates of the samples to show data coverage at different time points.

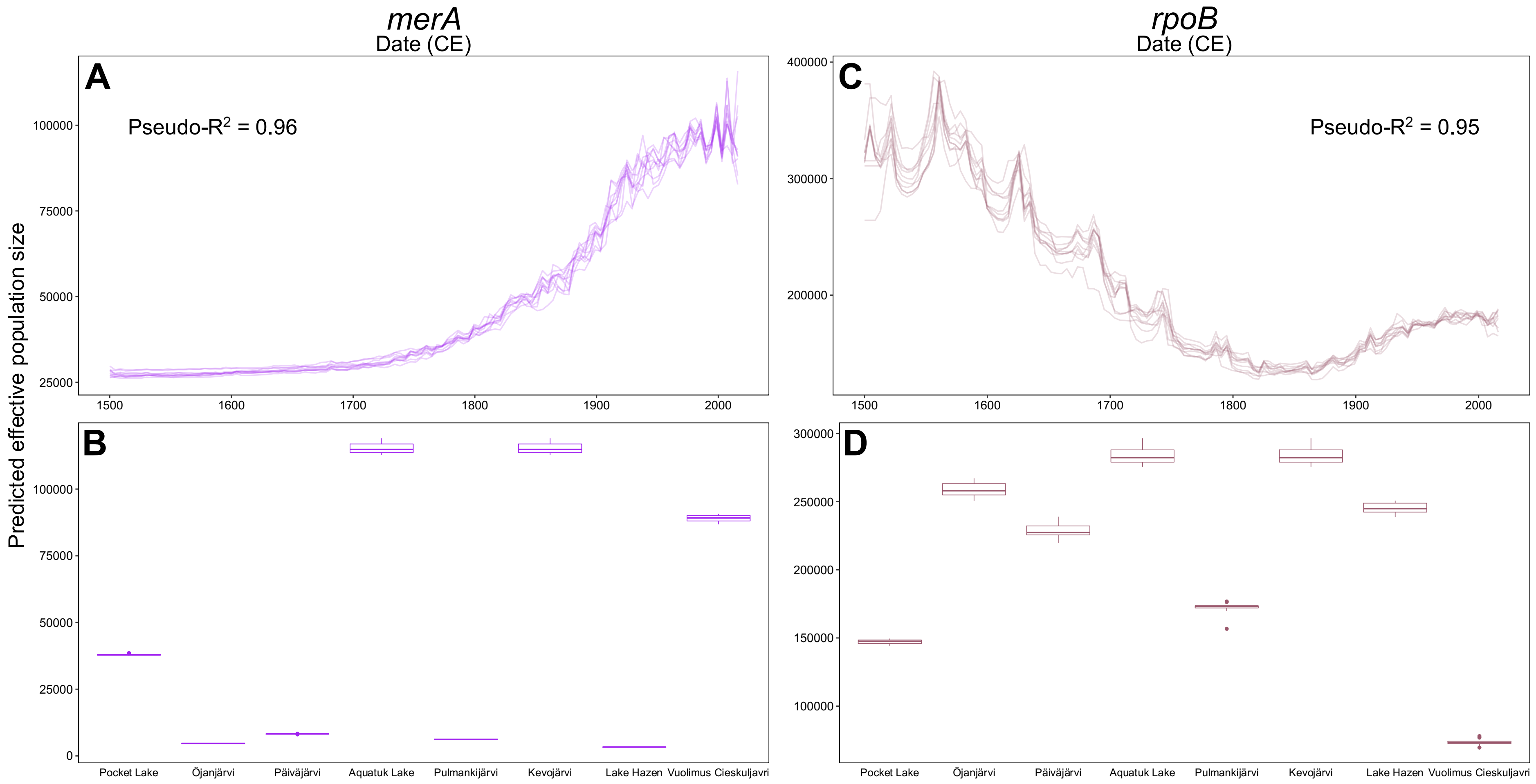

Figure S7. Partial dependence of predicted effective population sizes. Shown for *merA* (**A**, **B**) and *rpoB* (**C**, **D**) on their two predictors in the random forest models: CE date (**A**, **C**), and sampling site (**B**, **D**). Prediction accuracies are indicated in the top panels as pseudo-R^2. Each line in **A** and **B** and the variability per site shown in the box plots (**B**, **D**) shows the predictions of each individual model during the 10-fold cross-validation.

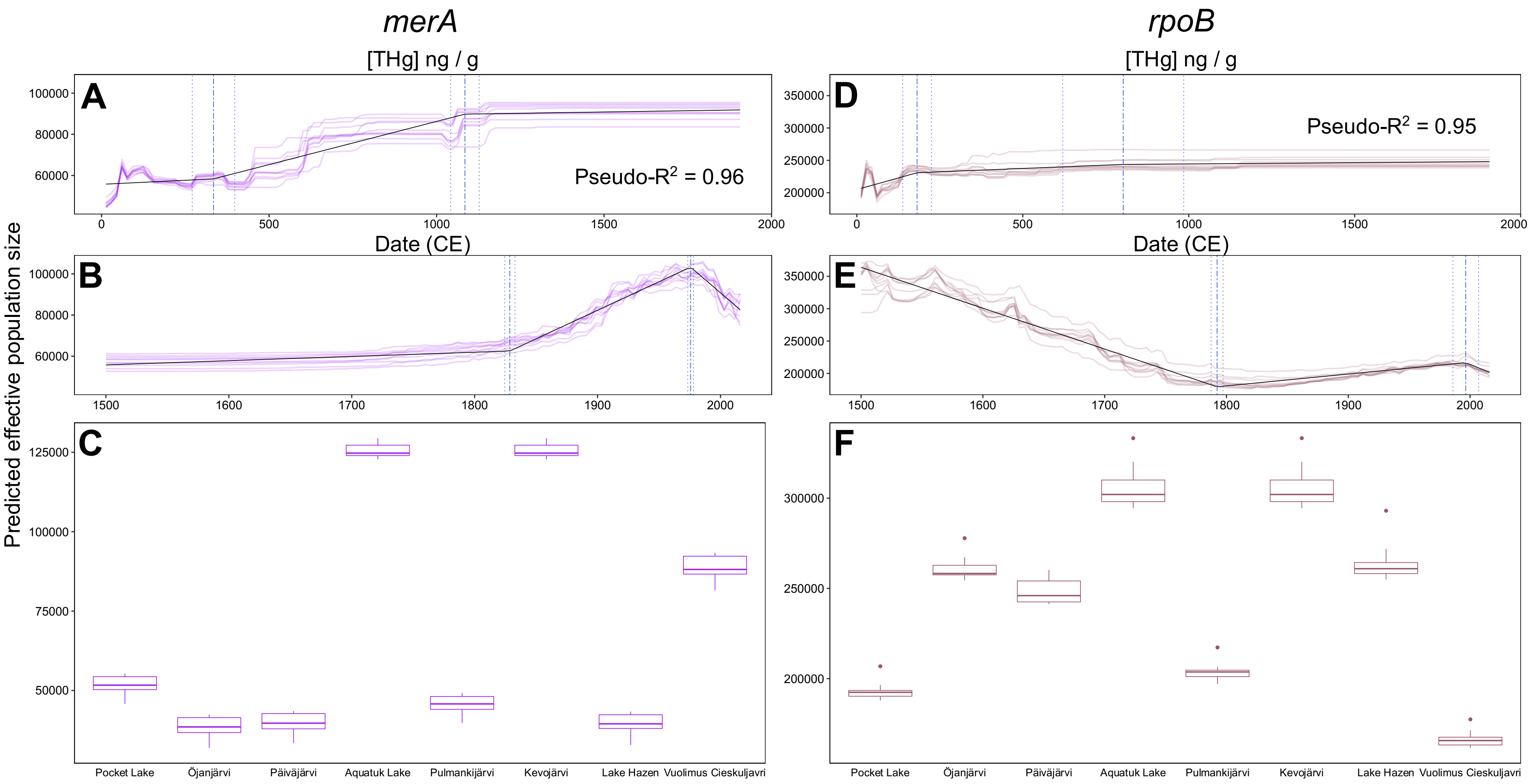

Figure S8. Partial dependence of predicted effective population sizes. Shown for *merA* (**A**, **B**, **C**) and *rpoB* (**D**, **E**, **F**) for the alternate random forest models, based on [THg] (**A**, **D**), CE date (**B**, **E**) and sampling site (**C**, **F**). Prediction accuracies are indicated in the top panels as pseudo-R^2. Each line in **A**, **B**, **D** and **E**, and the variability per site shown in the box plots (**C**, **F**) shows the predictions of each individual model in the 10-fold cross-validation. Three-part segmented linear models (black lines) were fit to the [THg] and date results for *merA* ([THg] $R^2 = 0.88$; date $R^2 = 0.95$) and *rpoB* ([THg] $R^2 = 0.46$; date $R^2 = 0.95$). The blue lines show the breakpoint estimates (dot-dash lines) and their 99% CIs (dotted lines).

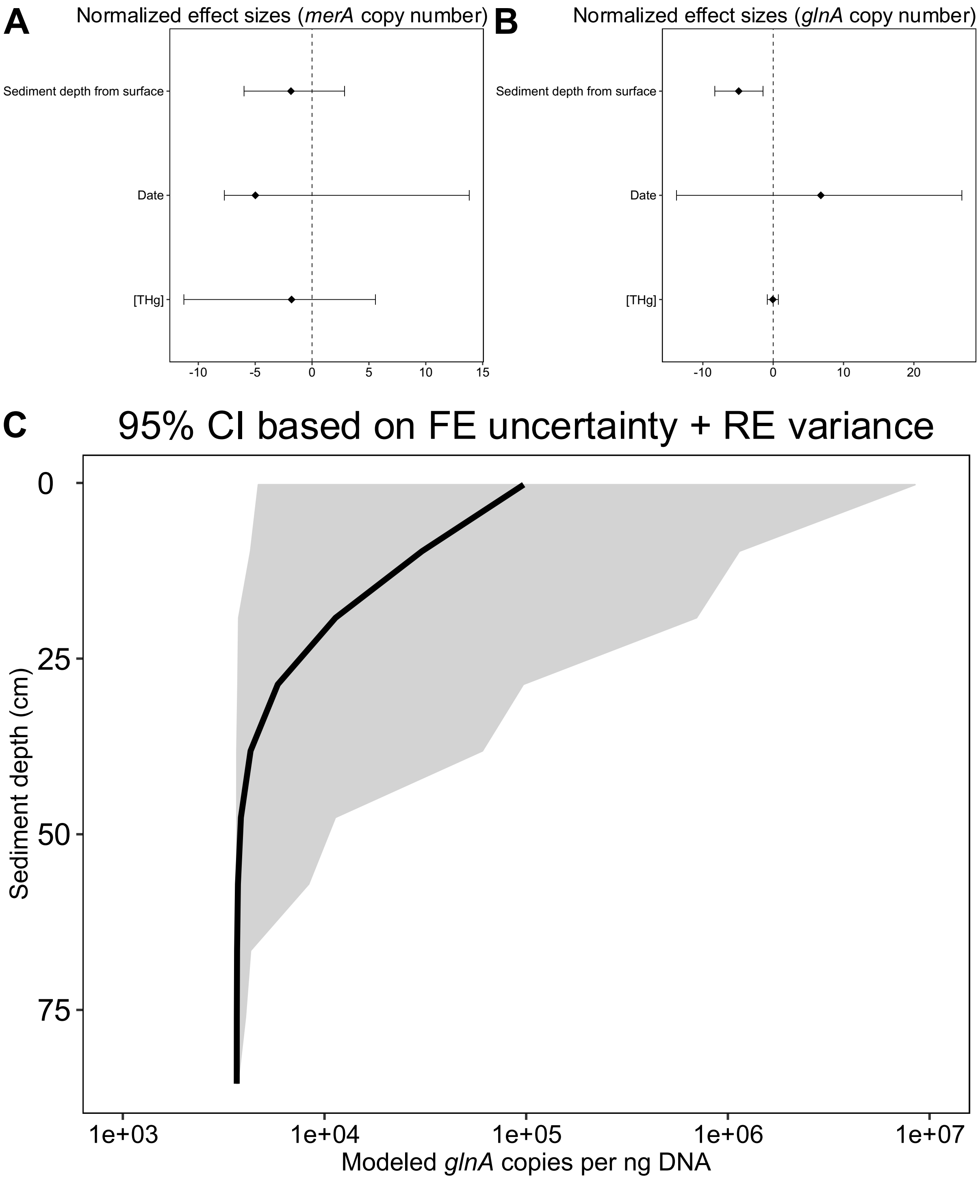

Figure S9. Drivers of gene abundances – droplet digital PCR analyses. Results of the mixed linear models showing the 95% confidence intervals of model terms for (**A**) *merA* and (**B**) *glnA* copy numbers on scaled and mean-centered variables, and (**C**) estimated effect size of sediment depth (distance from sediment surface) on the copy numbers of *glnA*. FE = Fixed Effect, RE = Random Effect.

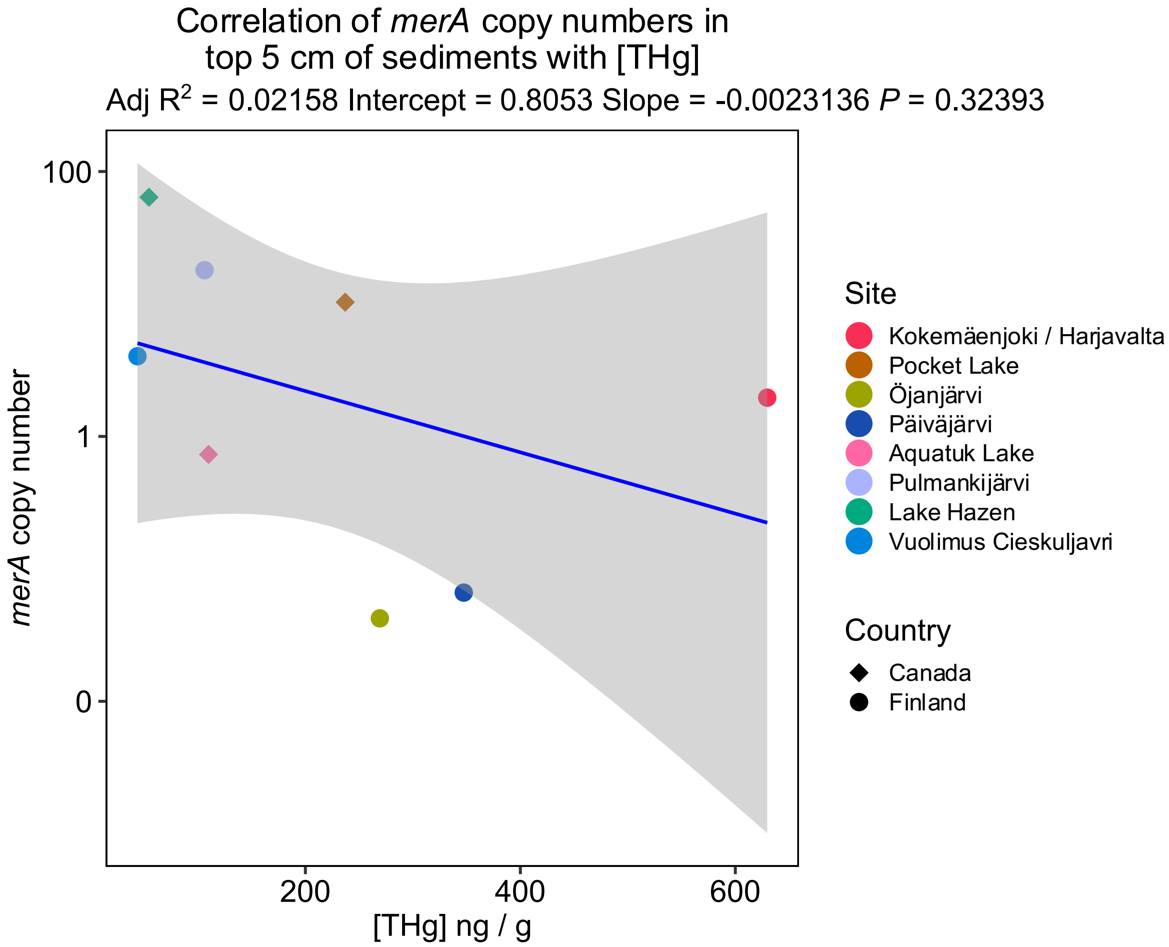

Figure S10. Gene abundance as a function of total mercury concentrations. Correlation between mean *merA* copy number and mean [THg] in the topmost 5 cm of sediment at each site. Least-square model fit is shown in blue, with its 95% confidence interval shown as the shaded region.

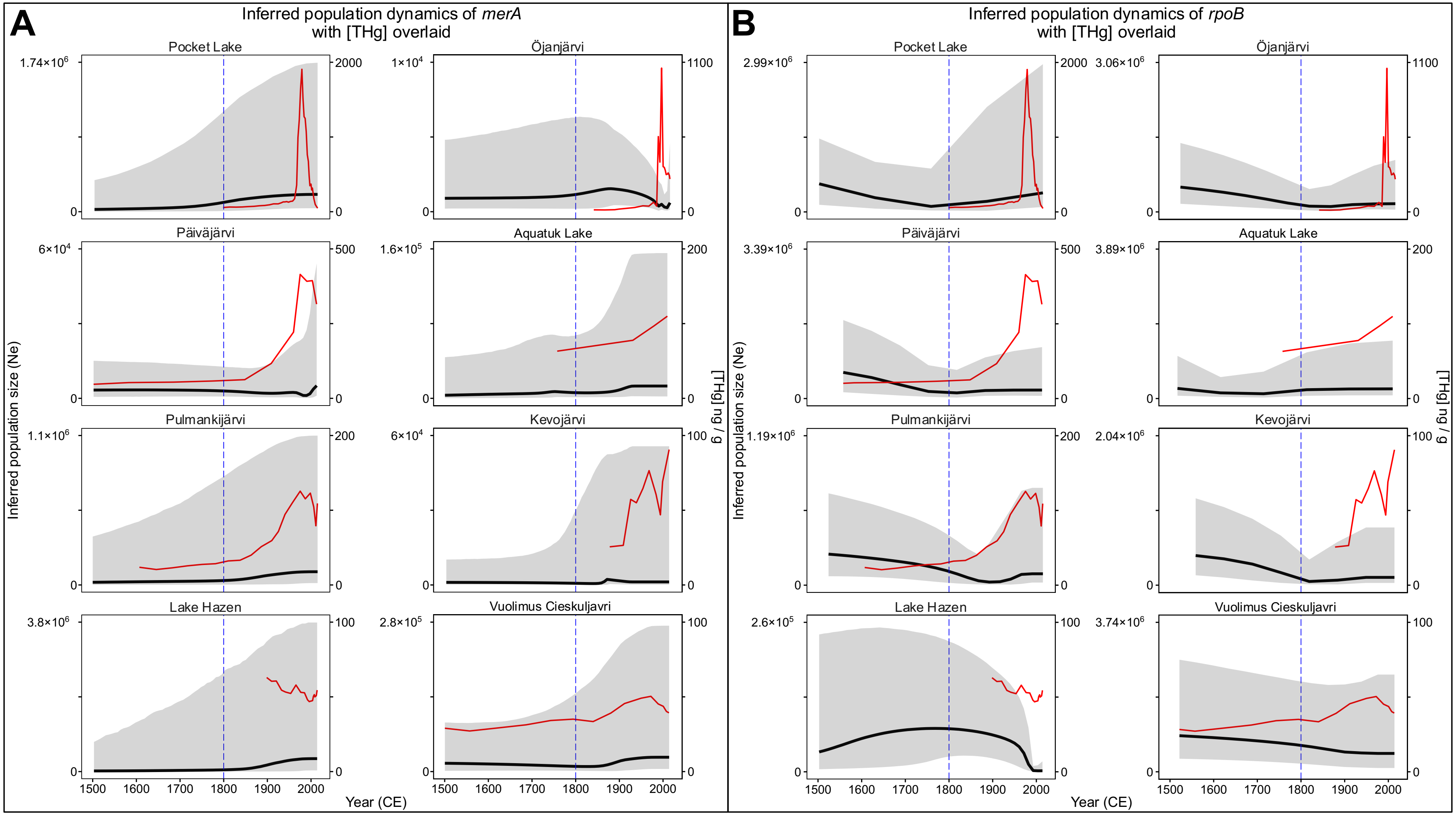

Figure S11. Demographic reconstructions. These are shown for *merA* (**A**) and *rpoB* (**B**) over calendar date with [THg] overlaid. In each panel, the black thick line shows the median of inferred effective population size and the grey area shows the 95% credible interval, the red line is the measured [THg], and the vertical blue dashed line indicates year 1800 CE, the approximate onset of the Industrial Revolution in the Northern Hemisphere.

Table S1. Gene quantification with droplet digital PCR and amplicon sequencing. PCR primer pairs used in the study

Target gene	Name	Used in	Sequence (5' – 3')	Product length	Master mix (per reaction)		PCR conditions	Reference
Mercuric reductase *(merA)*	qmerA1 F<br>qmerA1 R	ddPCR	CAT GAC GGT GCA GGA ACT G<br>GCT GCT TCA CAT CCT TGT TG	101 bp	QX200 MM (2x)<br>H_2O<br>10 µM FW/RV primers<br>Template<br>**Total**	11 µL<br>8.33 µL<br>(ea.) 0.33 µL<br>2 µL<br>**22 µL**	95°C 5 min<br>40x {95°C 30 s, 60°C 1 min}<br>4°C 5 min<br>90°C 5 min<br>10°C ∞	[53]
	NlfF<br>NlfR	Nested PCR (long product)	CCA TCG GCG GCA CYT GCG TYA A<br>CGC YGC RAG CTT YAA YCY YTC RRC CAT YGT	1247 bp	EconoTaq PLUS MM (2x)<br>H_2O<br>10 µM FW/RV primers<br>Template<br>**Total**	25 µL<br>19 µL<br>(ea.) 2.5 µL<br>1 µL<br>**50 µL**	94°C 2 min<br>25x {94°C 30s, 61°C 30s, 72°C 90s}<br>72°C 5 min<br>10°C ∞	[56]
	NsfF<br>NsfR	Nested PCR (short product)	ACA CTG ACG ACA TGG TTC TAC A<br>CGC YGC RAG CTT YAA YCY YTC RRC CAT YGT (same as NlfR)	308 bp	EconoTaq PLUS MM (2x)<br>H_2O<br>10 µM FW/RV primers<br>Template from NlfF-NlfR PCR<br>**Total**	12.5 µL<br>9 µL<br>(ea.) 1.25 µL<br>1 µL<br>**25 µL**	94°C 2 min<br>35x {94°C 30s, 61°C 30s, 72°C 30s}<br>72°C 5 min<br>10°C ∞	[56]
	NsfF_CS1<br>NlfR_CS2	Illumina MiSeq sequencing	ACA CTG ACG ACA TGG TTC TAC AAT CCG CAA GTN GCV ACB GTN GG<br>TAC GGT AGC AGA GAC TTG GTC TCG CYG CRA GCT TYA AYC YYT CRR CCA TYG T	352 bp	(Similar to NsfF – NsfR above)			[56] Common sequence 1 or 2 tags added as required by Illumina MiSeq
Glutamate synthetase *(glnA)*	GS1β<br>GS2γ	ddPCR	GAT GCC GCC GAT GTA GTA<br>AAG ACC GCG ACC TTY ATG CC	153-156 bp	QX200 MM (2x)<br>H_2O<br>10 µM FW/RV primers<br>1:10 diluted template<br>**Total**	11 µL<br>8.33 µL<br>(ea.) 0.33 µL<br>2 µL<br>**22 µL**	95°C 5 min<br>40x {95°C 30 s, 60°C 1 min}<br>4°C 5 min<br>90°C 5 min<br>10°C ∞	[54]
Ribosomal polymerase subunit B *(rpoB)*	rpoB_ssF<br>rpoB_ssR	PCR	CDG AAG GYC CRA ACA TYG<br>CYT GRC GYT GCA TGT TRG	375 bp	EconoTaq PLUS MM (2x)<br>H_2O<br>10 µM FW/RV primers<br>Template<br>**Total**	12.5 µL<br>9 µL<br>(ea.) 1.25 µL<br>1 µL<br>**25 µL**	94°C 2 min<br>35x {94°C 30s, 53°C 30s, 72°C 30s}<br>72°C 5 min<br>10°C ∞	(This study)
	rpoB_ssF_CS1<br>rpoB_ssR_CS2	Illumina MiSeq sequencing	ACA CTG ACG ACA TGG TTC TAC ACD GAA GGY CCR AAC ATY G<br>TAC GGT AGC AGA GAC TTG GTC TCY TGR CGY TGC ATG TTR G	419 bp	(Similar to rpoB_ssF – rpoB_ssR above)			(This study) Common sequence 1 or 2 tags added as required by Illumina MiSeq
